# Assessment of addiction management program and predictors of relapse among inpatients of the Psychiatric Institute at Ain Shams University Hospital

**DOI:** 10.1186/s43045-022-00246-5

**Published:** 2022-10-19

**Authors:** Nahla El Sayed Nagy, Eman Ibrahim Abo Ella, Eman Mohamed Shorab, Mohamed Hossam El-Din Abdel Moneam, Arwa Ahmed Tohamy

**Affiliations:** Okasha Institute of Psychiatry, Ain Shams University, Abassia, Ramses street extension, P.O. Box 11657, Dair AL-Malak, Cairo Egypt

**Keywords:** Addiction rehabilitation program, SUD, Relapse predictors, ASI, QOL

## Abstract

**Background:**

Rehabilitation programs targeted to patients with substance use disorder (SUD) following successful detoxification constitute a global public health concern. This study aimed to examine the effectiveness of a combined pharmacotherapy/cognitive behavior therapy (CBT) model through assessing abstinence/relapse rate and quality of life (QOL) in a sample of patients with SUD. Indeed, we aimed to identify the relapse predictors.

**Results:**

The relapse rate in the inpatient group was 45.33%, compared to 56% in the outpatient group. Multivariate analysis revealed that patients with educational levels less than secondary school, rural residency, being single or divorced, having cravings lasting for 6 weeks from detoxification, legal history, presence of borderline, antisocial and multiple personality disorder could predict relapse in patients with SUD. Moreover, there was a statistically significant difference between the legal, substance, and social domains of ASI (*X*^2^= 12.525, *p*=0.014; *X*^2^= 12.525, *p*=0.023; and *X*^2^= 6.335, *p*=0.042 respectively) and the majority of QOL domains and relapse.

**Conclusions:**

Socio-demographic data, legal history, craving, and presence of co-morbid personality disorders along with, legal, substance, and social domains of ASI might be implicated in relapse, suggesting that addiction rehabilitation programs targeting these topics would reduce the risk of relapse.

## Background

Substance use disorder is a devastating crisis in Egypt that has sounded alarm bells in both the society and government [1]. According to the United Nations Office on Drugs and Crime, 6–8% of adult Egyptians use cannabis [2]. According to the national addiction survey, Egyptians aged 20 to 45 were the most vulnerable age group, leading to an impact on educational, medical, and legal issues [3]. According to the Ministry of Health report on drug addiction in Cairo, 1.4 million people were addicted to drugs, particularly heroin and/or tramadol [4].

CBT, which integrates cognitive and behavioral theory, has been one of the most widely used techniques for decades [5]. CBT has been utilized to treat SUDs alone or in combination with pharmacological therapies [6], as well as other mental health comorbidities [7]. CBT for SUDs could enhance long-term abstinence through focusing on re-constructing cognitive errors about patients, others, and the environment, as well as engaging in coping skills training and rebuilding a balanced lifestyle [8].

Research work had addressed a great diversity in CBT sessions; the more CBT sessions would result in better performance and longer durations of abstinence for chronic substance users [9].

Relapse in SUD is widespread; it refers to a failure in a person’s attempt to change substance use behaviors, such as returning to pre-treatment drinking levels or continuing to use substances after a period of sobriety [10, 11].

Globally, relapse rates after treatment are high; it is higher in low- and middle-income countries compared to high-income countries [12]. In developing countries like South Africa, relapse has a negative impact on achieving the social development goals and places significant demands on social work service delivery [13].

Relapse rates for heroin [14] and other illicit drugs [15, 16] were estimated to be between 40 and 75% in European research. Relapse rates after treatment have been shown in other studies to be as high as 40–75% in the 3 weeks to 6 months following treatment [17].

Furthermore, researches had linked to a variety of factors to relapse in patients with SUD, which are categorized as individual, socio-demographic, psychiatric, medical diseases, and socio-cultural impacts [18]. Predictors of relapse might be as follows: young age at initiation, sex, unemployment, single status, peer group influence, family history of substance use, conflict and poor family support, and environmental factors such as drug availability and accessibility [11, 17, 19, 20].

Quality of life (QOL) is an important measurement and outcome in the management and treatment of chronic diseases, including SUDs [21]. In comparison to other medical professions, the SUD treatment sector has less carefully gathered and prioritized patient QOL [22]. Importantly, QOL measures incorporate patients’ subjective appraisals of the effects of SUD and its treatment on their life [23].

Patients with SUD had lower QOL compared to healthy populations. Hence, treatment should be tailored to individuals with these vulnerabilities to improve their QOL [21].

Consequently, we aimed in the current study to examine the effectiveness of a combined pharmacotherapy and cognitive behavior therapy (CBT) model through assessing the abstinence/relapse rate and quality of life (QOL) in a sample of patients with SUD. Indeed, the study aimed to identify the predictors for relapse. This study was based on the hypothesis that receiving an inpatient program followed by follow-up at the outpatient clinic for at least 6 months would be associated with less relapse, and it was hypothesized that specific sociodemographic variables, substance-related factors, and specific domains of ASI would predict relapse.

## Methods

### Participants

A total of 150 male patients with SUD divided into 75 patients from the inpatient wards (patient group) and 75 patients from the outpatient clinics (control group) were recruited from the Institute of Psychiatry, Ain Shams University Hospitals.

The sample size was calculated by reviewing the existing literature of similar studies (Andersson et al.) [24] using the PASS 11 program sample size calculation program. Determining the incidence rate of relapse in patients from the inpatient wards to be 30% + 10% and those following in the outpatient clinics to be higher than inpatients by 20%, a sample size of 75 patients per each group (total 150 patients) can detect this difference with the power of 80%.

The diagnosis of current SUD was made according to Structured Clinical Interview for DSM-IV Axis I Disorders (SCID-I) [25]. Participants were assessed through SCID-II to detect co-morbid personality disorder/s [26]. Severity of addiction was assessed through the Addiction Severity Index (ASI) [27]. Subsequently, participants performed the Physical component, Cognitive component, Affective component, Social component, Economic component, and Ego functioning (PCASEE) quality of life questionnaire [28].

Male patients with current SUD and have been admitted to the inpatient ward for at least 6 weeks, followed by a follow-up in the outpatient clinics for at least 6 months, and with ages ranging from 18 to 55 were enrolled in the current study after giving informed consent. Meanwhile, patients with any co-existing neuropsychiatric disorder and patients known to have an adverse reaction to naltrexone therapy were excluded.

The control group were recruited from the addiction outpatient clinics, with a history of current SUD, and at least a follow-up for 6 months not preceded by admission to the inpatient wards. They were demographically matched with the group of patients. Those with any co-existing neuropsychiatric disorder and patients known to have adverse reactions to naltrexone therapy were excluded.

### Procedure (all participants were subjected to the following)

#### Clinical assessment

Full history taking includes socio-demographic data as per the designed questionnaire [29], which identifies different clinical and demographic correlates to help in studying factors of relapse. It includes age, marital status, residency, employment status, income, education, and occupation. Details of substance use disorder were obtained including the duration of intake, daily dose, and periods of abstinence. Then, the psychometric assessment was conducted, after the detoxification period, as this would allow more stabilization.

#### Structured Clinical Interview for DSM-IV Axis I Disorders (SCID-I) [25]

The Arabic version of SCID I was previously validated and used in our study accordingly [30].

#### Structured Clinical Interview for DSM-IV Axis II Disorders (SCID-II) [26]

The Arabic version of SCID II was previously validated and used accordingly in our study [31].

#### The Addiction Severity Index (ASI) [27]

The ASI was intended for assessing problems presented by patients with SUDs, done before the engagement in the program. The Arabic version was used in this study [32].

#### PCASEE quality of life questionnaire [28]:

This is performed twice, before engagement in the program and after 6 months of follow-up. It provides information on symptoms and functioning over the last month. It is a 30-item self-rating scale completed on the basis of semi-structured interview. The Arabic version was used [33].

*The program for the patients’ group includes the following:*

- Detoxification: for a minimum of 7–10 days. During this period, the patient received pharmacotherapy in the form of analgesics, antidepressants, anxiolytics, antiepileptics, and sedatives according to the condition of each patient
- Psychometric assessment: by the previously mentioned tools (SCID-I, SCID II, ASI, and PCASEE)
- Rehabilitation: all patients went through individual sessions, twice per week, done by the researcher for 6 weeks. The following topics were covered: decisional balance sheet, causes of relapse and abstinence, craving table and functional analysis, internal and external cues, family dynamics, the key person, core belief, automatic thoughts, table of negative emotions, and discharge plan. Indeed, the patients went through a fixed 6-week schedule of group therapy sessions with the following structure; a morning meditation group (by a psychologist at 10 am), the main afternoon group (by a psychiatrist at 1:30 pm), another main group for practice/continuation of the afternoon group (by a psychiatrist at 4:30 pm), and a reflection group (by a psychiatrist at 6 pm). Each day has its specific topics as follows: *Saturday*: Addiction definition, neurobiology of addiction, medical complications, psychiatric complications, pharmacotherapy, and the law. *Sunday*: cognitive model, cognitive error, emotion log, anger, functional analysis of cravings, and irrelevant decisions. *Monday:* craving, internal triggers, spirituality, work and recovery, relationships, and vacations. *Tuesday*: the skills of asking, saying No, expressing emotions, answering embarrassing questions, validation, and apologizing. *Wednesday:* external triggers, truthfulness, accumulating positive emotions, illness, and recovery, making friends and high-risk situations. *Thursday* making decisions, problem-solving, daily schedule, setting SMART goals, barriers to relapse and mooring lines, and emergency plan [34, 35]
- Discharge: patients were discharged on symptomatic treatment tailored according to the patient’s condition. Patients diagnosed with opioid use disorder received naltrexone 50mg tab per day on discharge
- Follow-up in outpatient clinic for 6 months

*The program for the control group (after detoxification) and the patient group (after discharge) includes the following:*

- Regular weekly visits in the Saturday outpatient clinics for 2 months where the patients received individual sessions with the psychiatrist and the psychologist. After 2 months, the patients were transferred to Tuesday clinics where they received individual and group CBT sessions for 4 months. It is important to mention that during the era of COVID-19, patients were attending to the clinics just to receive their medications and to do the toxicological screening. Individual and group sessions were done using Tele psychiatric services
- During the inpatient stay and outpatient follow-up appointments, at least one attendant/caregiver was identified for each patient, who was usually a close family member who would stay with the patient. They were in charge of overseeing daily medication at home and were advised to write a note if they suspected their patient of any substance abuse relapse
- Urine sample for toxicological screening has been done every visit to monitor relapse for both groups. A positive test result was considered a relapse.
- Patients who dropped any follow-up were considered relapsed (for both patient and control groups)

### Statistical analysis

Data were analyzed through the Statistical Program for Social Science (SPSS) version 25. The following statistical tools were applied for adequate assessment and evaluation of our outcomes: means, standard deviations, chi-square test, independent *t* test, Mann-Whitney test (*U* test), Fisher’s exact test, and Wilcoxon signed-rank test. The *P* value was applied to indicate the level of significance where *P*≤0.001 is very highly significant, *P*≤0.01 is highly significant, and *P*≤0.05 is considered significant.

## Results

### Socio-demographic variables of the participants

The study included a total number of 150 SUD male patients, divided equally to the patient group (inpatients) and control group (outpatients). The studied groups were matched based on age, marital status, residence, education, and occupation with no statistically significant difference between the patient and control groups.

All participants were males aged between 18 and 55 years. Regarding residence, 44.7% live in rural areas while 55.3% live in urban ones. Concerning education, 32% of the patients did secondary education, and 10% of them were illiterate, while 19.3% was the percentage of patients who can read and write, who did preparatory education, and university graduates each. In terms of marital status, 47.3% were married, 40.7% were single, and 12% were divorced. Regarding occupation, 55.3% were employed while 44.7% were unemployed

There was a statistically significant difference between the patient group (inpatients) and control group (outpatients) regarding income; 35 (46.67%) of the control group had an income of more than 3000 LE, compared to 23 (30.67%) in the patient group (*X*^2^ = 4.048, *p*=0.044).

### Clinical characteristics of the participants

The commonest substance of abuse was heroin (71, 47.3%), followed by synthetic cannabinoids (26, 17.3%), and the least was alcohol (1, 0.7%). The highly reported duration of intake was more than 3 years (148, 98.7). The commonest route of intake was injection (49, 32.7%), followed by smoking (35, 23.3%) (Table 1).

**Table 1:** Clinical characteristics of the participants with regard to substance-related factors

		*N*	%
Main substance	Hash	8	5.3%
	Synthetic cannabinoids	26	17.3%
	Tramadol	18	12.0%
	Heroin	71	47.3%
	Amphetamine	1	0.7%
	Polysubstance	22	14.7%
	Pregabalin	3	2.0%
	Slcohol	1	0.7%
Max dose of heroin if it is the main substance	Less than 0.5	2	2.3%
	0.5–1 gm	26	30.2%
	More than 1 gm	58	67.4%
Route of intake	Smoke	35	23.3%
	Injection	49	32.7%
	Oral	22	14.7%
	Multiple	22	14.7%
	Sniffing	22	14.7%
Duration of abuse	1–3 years	2	1.3%
	More than 3 years	148	98.7%

### Predictors of relapse among both groups (relapsed and abstinent), with regard to sociodemographic characteristics

There was no statistically significant difference between the patient group and control group regarding relapse (45.33% and 56%, respectively) (*X*^2^= 1.707, *p*=0.191). On comparing relapsed patients to patients who could achieve abstinence for 6 months with a regular follow-up, it was found that relapse was associated with rural residency (*X*^2^= 18.088, *p*<0.001), low level of education (*X*^2^= 39.472, *p*<0.001), and being single or divorced (*X*^2^= 10.748, *p*=0.005 (Table 2)). These results were supported by performing regression analysis; the multivariate analysis showed that the level of education (secondary or above) (*p* <0.001, OR=13.65) and being married (*p*= 0.034, OR=3.15) could predict relapse/abstinence (Table 4).

**Table 2:** Predictors of relapse among both groups (relapsed and abstinent), with regard to the sociodemographic characteristics

		Relapse group	Abstinent group			
		*N*=76 (50.7%)	*N*=74 (49.3%)	Value	*p* value	Sig.
Age	Below 20	4 (5.26%) ^a^	0 (0%) ^b^	Fisher’s Exact test	0.041	S
	20–40	56 (73.68%) ^a^	64 (86.49%) ^a^			
	>40	16 (21.05%) ^a^	10 (13.51%) ^a^			
Residence	Urban	21 (27.63%)	46 (62.16%)	***X***^***2***^***=*** 18.088	<0.001	S
	Rural	55 (72.37%)	28 (37.84%)			
Education	Illiterate	12 (15.79%) ^a^	3 (4.05%) ^b^	***X***^***2***^***=*** 39.472	<0.001	S
	Reads and writes	23 (30.26%) ^a^	6 (8.11%) ^b^			
	Preparatory	21 (27.63%) ^a^	8 (10.81%) ^b^			
	Secondary school	14 (18.42%) ^a^	34 (45.95%) ^b^			
	University graduate	6 (7.89%) ^a^	23 (31.08%) ^b^			
Marital status	Single	38 (50%) ^a^	23 (31.08%) ^b^	***X***^***2***^***=*** 10.748	0.005	S
	Married	26 (34.21%) ^a^	45 (60.81%) ^b^			
	Divorced	12 (15.79%) ^a^	6 (8.11%) ^a^			
Occupation	Not working	39 (51.32%)	28 (37.84%)	***X***^***2***^***=*** 2.756	0.097	NS
	Working	37 (48.68%)	46 (62.16%)			
Income	Less than 3000	43 (56.58%)	49 (66.22%)	***X***^***2***^***=*** 1.468	0.226	NS
	More than3000	33 (43.42%)	25 (33.78%)			

*P* value > 0.05: significant; *S* significant, *NS* non-significant, *N* number

Each superscript letter (a and b) denotes a subset of Group categories whose column proportions do not differ significantly from each other at the .05 level

### Predictors of relapse among both groups (relapsed and abstinent), with regard to substance-related factors

There was a statistically significant difference between relapsed patients and patients who could achieve abstinence for 6 weeks with regular follow-up regarding the main substance of abuse (*p*=0.002), route of intake (*X*^*2*^*=* 13.297, *p*=0.01), presence of craving for the substance after 6 months (*X*^*2*^*=*16.577, *p*<0.001), previous attempts of abstinence (*X*^*2*^*=*15.87, *p*<0.001), presence of legal history (*X*^*2*^*=*13.998, *p*<0.001), and history of imprisonment (*X*^*2*^*=*6.802, *p*=0.009) (Table 3). These results were supported by performing regression analysis; the multivariate analysis showed that craving after 6 weeks (*p*=0.004, OR=0.09), and legal history (*p*=0.004, OR=0.19) could predict relapse/abstinence (Table 4).

**Table 3:** Predictors of relapse among both groups (relapsed and abstinent), with regard to substance-related factors

		Relapsed group	Abstinent group			
		*N*=76 (50.7%)	*N*=74 (49.3%)	Test	*p* value	Sig.
Main substance	Hash	2 (2.63%) ^a^	6 (8.11%) ^a^	Fisher’s Exact test	0.002	S
	Synthetic cannabinoids	15 (19.74%) ^a^	11 (14.86%) ^a^			
	Tramadol	4 (5.26%) ^a^	14 (18.92%) ^b^			
	Heroin	37 (48.68%) ^a^	34 (45.95%) ^a^			
	Amphetamine	0 (0%) ^a^	1 (1.35%) ^a^			
	Multiple	17 (22.37%) ^a^	5 (6.76%) ^b^			
	Pregabalin	0 (0%) ^a^	3 (4.05%) ^a^			
	Alcohol	1 (1.32%) ^a^	0 (0%) ^a^			
Max dose of heroin if it is the main substance	Less than 0.5	0 (0%)	2 (5.26%)	Fisher’s Exact test	0.34	NS
	0.5–1 gm	14 (29.17%)	12 (31.58%)			
	More than 1 gm	34 (70.83%)	24 (63.16%)			
Route of intake	Smoke	17 (22.37%) ^a^	18 (24.32%) ^a^	***X***^***2***^***=*** 13.297	0.01	S
	Injection	25 (32.89%) ^a^	24 (32.43%) ^a^			
	Oral	5 (6.58%) ^a^	17 (22.97%) ^b^			
	Multiple	17 (22.37%) ^a^	5 (6.76%) ^b^			
	Sniffing	12 (15.79%) ^a^	10 (13.51%) ^a^			
Duration of abuse	1–3 years	0 (0%)	2 (2.7%)	Fisher’s Exact test	0.242	NS
	More than 3 years	76 (100%)	72 (97.3%)			
Craving after 6weeks	No	8 (10.53%)	29 (39.19%)	16.577	<0.001	S
	Yes	68 (89.47%)	45 (60.81%)			
Previous attempts of abstinence	0	6 (7.89%) ^a^	19 (25.68%) ^b^	15.87	<0.001	S
	1	34 (44.74%) ^a^	40 (54.05%) ^a^			
	Two or more	36 (47.37%) ^a^	15 (20.27%) ^b^			
Legal history	No	36 (47.37%)	57 (77.03%)	13.998	<0.001	S
	Yes	40 (52.63%)	17 (22.97%)			
Imprisonment	No	55 (72.37%)	66 (89.19%)	6.802	0.009	S
	Yes	21 (27.63%)	8 (10.81%)			

*P* value > 0.05: significant; **S** significant, *NS* non-significant, *N* number

Each superscript letter (a and b) denotes a subset of Group categories whose column proportions do not differ significantly from each other at the .05 level

**Table 4:** Regression analysis to the predictors of relapse (sociodemographic, substance-related factors, and SCID-II) among both groups (relapsed and abstinent)

	Univariate analysis		Multivariate analysis	
	*p* value	OR (95% CI)	*p* value	OR (95% CI)
Education (secondary or above)	<0.001	9.39 (4.46–19.76)	<0.001	13.65 (4.27–43.59)
Marital status (married)	0.001	2.98 (1.53–5.8)	0.034	3.15 (1.09–9.11)
Tramadol	0.01	4.2 (1.31–13.44)	0.928	0.79 (0–134.11)
Multiple drugs	0.007	0.25 (0.09–0.72)	0.249	0.39 (0.08–1.93)
Oral	0.005	4.24 (1.47–12.18)	0.966	1.12 (0.01–162.61)
Craving after 6weeks	<0.001	0.18 (0.08–0.44)	0.004	0.09 (0.02–0.47)
Legal history	<0.001	0.27 (0.13–0.54)	0.004	0.19 (0.06–0.59)
Narcissistic	0.037	3.8 (1–14.42)	0.409	2.14 (0.35–13.07)
Borderline	0.021	0.38 (0.17–0.88)	0.039	0.25 (0.07–0.93)
Antisocial	0.001	0.14 (0.04–0.49)	0.031	0.09 (0.01–0.8)
Multiple personality disorders	0.01	0.2 (0.06–0.75)	0.001	0.05 (0.01–0.28)

*P value > 0.05: significant*

### Predictors of relapse among both groups (relapsed and abstinent), with regard to SCID-II

On performing regression analysis, our results indicated that having certain personality disorders namely borderline (*p*= 0.039, OR= 0.25), antisocial (*p*= 0.31, OR=0.09), and multiple personality disorder (*p*= 0.001, OR=0.05) could predict relapse/abstinence (Table 4).

### Predictors of relapse among both groups (relapsed and abstinent), with regard to ASI

There was a statistically significant difference between relapsed patients and patients who could achieve abstinence for 6 months with a regular follow-up regarding legal *(X*^*2*^*=* 12.525, *p*=0.014), substance (*p*=0.023), and social *(X*^*2*^*=* 6.335, *p*=0.042) domains of ASI (Table 5).

**Table 5:** Predictors of relapse among both groups (relapsed and abstinent), with regard to ASI

		Relapsed group	Abstinent group	Test of significance		
		*N*=76 (50.7%)	*N*=74 (49.3%)	Test	*p* value	Sig.
ASI- Medical	No problem	0 (0%)	1 (1.35%)	Fisher’s Exact test	0.522	NS
	Slight problem	43 (56.58%)	42 (56.76%)			
	Moderate problem, some ttt indicated	6 (7.89%)	9 (12.16%)			
	Considerable problem, ttt necessary	2 (2.63%)	0 (0%)			
	Extreme problem, ttt absolutely necessary	25 (32.89%)	22 (29.73%)			
ASI –Legal	No problem	39 (51.32%) ^a^	56 (75.68%) ^b^	***X***^***2***^***=*** 12.525	0.014	S
	Slight problem	3 (3.95%) ^a^	1 (1.35%) ^a^			
	Moderate problem, some ttt indicated	6 (7.89%) ^a^	5 (6.76%) ^a^			
	Considerable problem, ttt necessary	9 (11.84%) ^a^	7 (9.46%) ^a^			
	Extreme problem, ttt absolutely necessary	19 (25%) ^a^	5 (6.76%) ^b^			
ASI –occupational	No problem	0 (0%)	1 (1.35%)	Fisher’s Exact test	0.475	NS
	Slight problem	0 (0%)	1 (1.35%)			
	Moderate problem, some ttt indicated	13 (17.11%)	15 (20.27%)			
	Considerable problem, ttt necessary	26 (34.21%)	29 (39.19%)			
	Extreme problem, ttt absolutely necessary	37 (48.68%)	28 (37.84%)			
ASI-Substance	Moderate problem, some ttt indicated	1 (1.32%) ^a^	5 (6.76%) ^a^	Fisher’s Exact test	0.023	S
	Considerable problem, ttt necessary	30 (39.47%) ^a^	40 (54.05%) ^a^			
	Extreme problem, ttt absolutely necessary	45 (59.21%) ^a^	29 (39.19%) ^b^			
ASI-social	Moderate problem, some ttt indicated	13 (17.11%) ^a^	26 (35.14%) ^b^	***X***^***2***^***=*** 6.335	0.042	S
	Considerable problem, ttt necessary	50 (65.79%) ^a^	38 (51.35%) ^a^			
	Extreme problem, ttt absolutely necessary	13 (17.11%) ^a^	10 (13.51%) ^a^			
ASI-Psychiatric	No problem	0 (0%)	1 (1.35%)	Fisher’s Exact test	0.208	NS
	Slight problem	35 (46.05%)	43 (58.11%)			
	Moderate problem, some ttt indicated	31 (40.79%)	20 (27.03%)			
	Considerable problem, ttt necessary	10 (13.16%)	10 (13.51%)			

*P* value > 0.05: significant; *S* significant, **NS** non-significant, *N* number, *ASI* addiction severity index, *ttt* treatment

Each superscript letter (a and b) denotes a subset of Group categories whose column proportions do not differ significantly from each other at the .05 level

### Predictors of relapse among both groups (relapsed and abstinent), with regard to QOL

There was a statistically significant difference between relapsed patients and patients who could achieve abstinence for 6 months with a regular follow-up before the start of the program, as we found that the abstinent group has higher grades in the QOL in cognitive (*z*= −4.171, *p*= <0.001), affective (*z*= −3.763, *p*= <0.001), and financial (*z*= −2.921, *p*= <0.003) domains in comparison to relapsed ones.

On comparing relapsed patients to abstinent ones after receiving the program, there was a significant improvement in all domains of QOL, physical (*z*= −10.863, *p*= <0.001), cognitive (*z*= −10.702, *p*= <0.001), affective (*z*= −10.692, *p*= <0.001) social (*z*= −10.651, *p*= <0.001), economic (*z*= −10.393, *p*= <0.001) and ego domain (*z*= −10.700, *p*= <0.001) (Table 6).

**Table 6:** Comparison between relapsed and abstinent groups in QOL before and after program

		Relapse group	Abstinent group	Mann-Whitney test		
		Median (IQR)	Median (IQR)	***Z***	*p* value	Sig.
Before the program	PCASEE-p	1 (0–7)	2 (0–8)	−0.994	0.320	NS
	PCASEE-C	6 (2.5–7.5)	9 (6–11)	−4.171	<0.001	S
	PCASEE-A	4 (2–7)	7 (4–9)	−3.763	<0.001	S
	PCASEE-S	6 (2–8)	7 (3–10)	−1.910	0.056	NS
	PCASEE-F	4.5 (0–8.5)	7 (4–12)	−2.921	0.003	S
	PCASEE-E	7 (5–10)	7 (5–10)	−0.381	0.703	NS
After the program	PCASEE-P	1 (0–7)	25 (24–25)	−10.863	<0.001	S
	PCASEE-C	6 (2.5–7.5)	25 (23–25)	−10.702	<0.001	S
	PCASEE-A	4 (2–7)	25 (22–25)	−10.692	<0.001	S
	PCASEE-S	6 (2–8)	25 (21–25)	−10.651	<0.001	S
	PCASEE-F	4.5 (0–8.5)	22 (20–25)	−10.393	<0.001	S
	PCASEE-E	7 (5–10)	25 (23–25)	−10.700	<0.001	S

*P* value > 0.05: significant; *S* significant, *NS* non-significant, *P* physical component, *C* cognitive component, *A* affective component, *S* social component, *E* economic component, and *E* ego functioning

## Discussion

The current study aimed to assess addiction management program at the institute of psychiatry in Ain Shams University, and this has been done through studying the relapse rate and effect of abstinence on QOL.

### Regarding the relapse rate in our study

The relapse rate was 45.33% in the inpatient group in comparison to patients following up in outpatient clinics where the relapse rate was 56% after 6 months of follow-up. This relapse rate is compatible with that reported in different studies. Bradizza et al. study revealed that more than 50% of persons with SUD relapsed after treatment [36]. Relapse rates after treatments are high according to different studies [17] and often reach 40–75% in 3-week to 6-month period following the therapy [37].

Studies carried out in several nations with high rates of inpatient treatment completion reveal a significant prevalence of relapse, including 33% in Nepal, 55.8% in China, and 60% in Switzerland [19].

These outcomes were relatively high when compared to a study of patients admitted to an inpatient stay in Norway, where 37% of the sample relapsed after 3 months. However, due to the diversity of patient demographics, treatment settings, and varied follow-up intervals and definitions of relapse, direct comparisons of relapse rates between studies are difficult [24].

The study explored the predictor risk factors of relapse among the patients diagnosed with SUD, and this has been done through comparing relapsed patients and patients who could achieve abstinence for 6 months with a regular follow-up regarding sociodemographic characteristics, substance-related factors, personality disorders, and addiction severity index domains.

### Regarding the clinical characteristics of the participants

The commonest substance of abuse was heroin (71, 47.3%) and followed by synthetic cannabinoids (26, 17.3%), and the least was alcohol (1, 0.7%). This was in agreement with Chalana et al. [29] study where heroin was the most preferred opioid of abuse. According to the United Nations Office on Drugs and Crime 2019 [38], following Nigeria, and Egypt reported the capture of the second-largest quantity of synthetic opioids.

This finding is consistent with the study conducted in Mansoura by El-Awady and his colleagues reporting that the most frequently used substance was tramadol [39].

However, our results are not in agreement with Hamdi et al. [40] finding that Cannabis was used by 77% of drug users. In all Egyptian governorates, with the exception of Upper Egypt, alcohol (28.6% of total consumption) was the second most commonly used drug (where opiates were commoner than alcohol). Opiates were the third most widely used drug in Egypt in governorates outside of Upper Egypt (23.4% of total use).

In a more recent study by Naguib et al. [41], the results revealed that hashish (96.5%), Strox (41.3%), Bhang (34.4%), Voodoo (34.4%), and Tramadol (31.1%) were the most commonly utilized drugs.

This difference in results could be due to the notice that patients with heroin and synthetic cannabinoids use disorder seek help at the institute of psychiatry after multiple trials in outer private clinics.

The most often used substance over the past 12 months and over the course of one’s lifetime was nicotine (9%) according to a 2020 study on the frequency of substance use among Egyptian adolescents. The most often abused substance after nicotine was excluded was benzodiazepines (5.1%), followed by alcohol (3.3%) and organic solvents (3.1%). Alcohol (2.9%), followed by chemical solvents (2.7%), and cannabis (2.6%), was the substance most frequently used over the previous 12 months. This difference may be due to the different age groups [42].

Regarding the route of intake, the commonest in this study was injection (49, 32.7%), followed by smoking (35, 23.3%), and this is matched with Chalana et al.’s [27] study but inconsistent with Saboula et al.’s study at Menoufia University Hospital where oral drug consumption accounted for more than half (52.5%) of the addicts during the study [43]. However, this will differ according to the substance mostly used in the study.

In this study, the highly reported duration of intake was more than 3 years (148, 98.7) and this is compatible with Kumar et al.’s study [44] which found that the duration of substance abuse was 4.66±4.52 years.

### Regarding predictors of relapse among both groups (relapsed and abstinent groups) with regard to sociodemographic characteristics

There was no statistically significant difference between the patient group and control group regarding relapse (45.33% and 56%, respectively) (*X*^2^= 1.707, *p*=0.191).

Researchers conducted to study outpatient management are scarce compared to studies including inpatients [45]. Our result is slightly higher than Daigre et al.’s [45] result where he found that the relapse rate in outpatients after 6 months of follow-up is 47.1%.

Bottlender et al. found that the relapse rate after completion of an intensive outpatient treatment program for alcoholism is 57% [46].

Although there is no clear data about the impact of receiving a combined inpatient and outpatient program on relapse rate in comparison to receiving the outpatient program only, a study showed a lower chance of relapsing for those who had fulfilled the inpatient stay. This is consistent with other research that emphasized the significance of completing SUD treatment for later drug use outcomes [47].

Non-significance in the current study may be explained by affection and change in the services provided during the era of COVID-19.

#### Residency

On comparing relapsed patients to patients who could achieve abstinence for 6 months with regular follow-up, it was found that relapse was associated with rural residency (*X*^2^= 18.088, *p*<0.001). Almost all of these patients who live in rural areas have low monthly income, according to Cerda et al. study. Individuals in this social stratum are frequently subjected to insufficient food, substandard housing, high levels of violence, and poor mental health, all of which may encourage them to use illicit drugs and engage in dangerous behaviors [48, 49]. Those were 53% in a Mouritanian study with a significant relation to relapse [50].

4.7% of substance users resided in urban areas. The majority of Bedouins in Delta and Upper Egypt used drugs. The least likely to use drugs were those with rural residency(s) [40]. Cultural and geographical differences may be the cause of this disparity.

#### Education

As for educational level and relapse, this study showed a significant relationship between relapse/abstinence and level of education by regression analysis (*p* value <0.001, OR 13.65 (4.27–43.59), and these results are matching with a study done by Kenneth et al. and his colleagues finding that of 114 patients with less than high school completed or a high school diploma, relapse rates were 50% and 33%, respectively. In contrast, of 76 patients with an associate, bachelor, or graduate degree, the relapse rates were 23%, 14%, and 18%, respectively. There was a strong linear-by-linear association [51]. And this aligns too with the study done by Xie et al. finding that relapse is associated with less than high school education [52].

There are only few researches that have different findings from the study of Kabisa et al., which found no relationship between education and relapse. As his sample included more than 50% of patients who completed secondary education [53], this may be because the study’s sample was different.

#### Marital status

Regarding marital status, the results documented that being married is associated with relapse/abstinence by regression analysis (*p* value 0.03, OR 3.15 (1.09–9.11)), our results are aligned with Stickley and colleagues stating that people seek “alternative gratifications” in attempt to cope with or lessen the negative feelings that might arise from loneliness, which may include dangerous behaviors such as drug usage [54].

However, inadequate support with respect to the family structure has also been identified as a risk factor for SUD [55]. The study results are inconsistent with the study done by Kabisa et al. where there was no significant relationship between marital status and relapse; meanwhile, the same study referred to the importance of family and parental support to maintain abstinence [53].

### Regarding predictors of relapse among both groups (relapsed and abstinence) with regard to substance-related factors

#### Main substance of abuse

There was a statistically significant difference between relapsed patients and patients who could achieve abstinence for 6 months with a regular follow-up regarding the main substance of abuse (*p*=0.002). Although findings revealed that tramadol abuse is more associated with abstinence and multiple drug abuse is in association with relapse, a regression study revealed that the type of drug abused and drug polyconsumption had no significant relationship with relapse, implying that recurrence was unrelated to the number of substances used. This outcome was consistent with Ramo et al.’s research [56], the Mauritian study [50], and Chalana et al.’s study [27]. These results are inconsistent with the study done in Icyzere, Rowanda, finding that patients with polysubstance use were more likely to relapse than those who only used one substance [53].

#### Craving

Regarding craving, the multivariate analysis showed that craving after 6 weeks (*p*=0.004, OR=0.09) could predict relapse/abstinence, and this is supported by Doweiko et al. stating that craving itself is a poor predictor of abstinence. It may be triggered by drug-use cues (smells, the sight of the drug, sounds, etc.), and it could trigger moods and memories that predispose the individual to drug use [57]. Our results are matching to a study done by Chalana et al. [27] and another study by Swanpoel et al. among young African adults [11].

#### Previous attempts for detoxification

Regarding previous attempts for detoxification, the study revealed that patients with the past attempt of substance detoxification had decreased the possibility of maintaining remission for a 6-month follow-up. These results were matching with Chalana et al.’s study [11] and Elkashef et al.’s study where the number of admissions per patient each year was used as an estimate of relapse [58].

In this study, results are compatible with studies finding that the most significant predictor was the number of previous detoxifications, which is an intuitive and influential outcome predictor [59, 60].

#### Legal history

Regarding legal history and imprisonment, the multivariate analysis in this study of legal history (*p*=0.004, OR=0.19) could predict relapse/abstinence; this is compatible with Chamberlain et al.’s [61] study where the multivariable regression model shows a significant association between post-release illicit substance use and time incarcerated during the most recent prison term. Our result is consistent with the findings of Chalana et al.’s [27] and Hubicka et al.’s study too [62].

### Regarding predictors of relapse among both groups (relapsed and abstinent), with regard to SCID-II

On performing regression analysis, results indicated that having certain personality disorders namely borderline (*p*= 0.039, OR= 0.25), antisocial (*p*= 0.31, OR=0.09), and multiple personality disorder (*p*= 0.001, OR=0.05) could predict relapse/abstinence. Prior to relapse, a substantial link was found between impulsivity and impaired thinking processes, showing that relapse is impulsive and that most addicts never think about the effort they put in during remission [63, 64] impulsivity is a trait observed in individuals with personality disorders such as antisocial personality disorder (ASPD) or borderline personality disorder (BPD) [65–67]. Results of the study are matching with a study which showed that up to 23% and 53% of patients suffering from ASPD and BPD, respectively, are strongly associated to SUD. Other studies revealed that patients with co-morbid personality disorders have a poor prognosis and a higher relapse rate [50].

### Regarding predictors of relapse among both groups (relapsed and abstinent), with regard to ASI

It is expected that patients with less addiction severity and who began treatment and abstinent have better outcomes [68], and this is exactly what we discovered in our study; the results showed that relapse is associated with increased severity of addiction in the legal (*X*^*2*^*=* 12.525, *p*=0.014), substance (*p*=0.023), and social *(X*^*2*^*=* 6.335, *p*=0.042) domains, which is consistent with a previous meta-analysis that found that the most powerful predictor of lower levels of QOL at admission to SUD treatment was the perceived severity of substance abuse [69].

In a study by Hubicka et al. [62], men who relapsed in driving under influence (DUI) had significantly (*p* ¼ 0.050) worse family and social relations, but did not differ in the other ASI domains.

### Regarding predictors of relapse among both groups (relapsed and abstinent), with regard to QOl

There was a statistically significant difference in QOL score before engagement to the program between relapsed patients and patients who could achieve abstinence for 6 months with a regular follow-up, as results showed that the abstinent group has higher grades in the QOL in cognitive (*z*= −4.171, *p*= <0.001), affective (*z*= −3.763, *p*= <0.001), and financial (*z*= −2.921, *p*= <0.003) domains in comparison to relapsed ones. Results are not in agreement with Picci et al.’s study whose primary goal was to evaluate the WHOQOL domains’ potential for prediction. The study discovered that none of the baseline QOL measures, however, were effective predictors of either relapse within 12 months of discharge or the intensity of alcohol use in relapsed patients. This difference in results may be due to the difference in follow-up duration [70].

### Assessment of addiction management program using QOL scale

This study aimed to assess the addiction management program at the Institute of Psychiatry at Ain Shams University, and this has been done through studying the relapse rate as discussed before and the effect of abstinence on QOL.

On comparing relapsed patients to abstinent ones after receiving the program, there was a significant improvement in all domains of QOL, physical (*z*= −10.863, *p*= <0.001), cognitive (z= −10.702, *p*= <0.001), affective (z= −10.692, *p*= <0.001), social (*z*= −10.651, *p*= <0.001), economic (*z*= −10.393, *p*= <0.001), and ego domain (*z*= −10.700, *p*= <0.001).

These results are in line with Vederhus et al.’s study identifying abstinence as a positive predictor of QOL [71] and with Tracy et al.’s study [72] and with Manning et al.’s study finding that improvements in QOL were considerable across all four domains among treatment responders. The improvements in the physical, psychological, social, and environmental domains’ scores were equivalent to improvements in the general population SDs of 0.73, 1.07, 0.92, and 0.75, respectively [73].

## Conclusions

In summary, identifying predictors of relapse in SUD patients has a crucial role in improving the addiction management program outcome. Moreover, we have to extend our target for more than abstinence, we have to target functional recovery and quality of life improvement.

### Limitations

- The study duration included the era of COVID-19 which affected the services provided to the patients.
- The study focused on one institute, so other centers were not included in the comparison.
- Study was limited to the target population that did not permit the researchers to generalize at the national level.

## Data Availability

Not available.

## Abbreviations

ASI: Addiction severity index
ASPD: Antisocial personality disorder
BPD: Borderline personality disorder
CBT: Cognitive behavioral therapy
DSM-IV: Diagnostic and Statistical Manual of Mental Disorders
PCASEE: (P) Physical component, (C) Cognitive component, (A) Affective component, (S) Social component, (E) Economic component, and (E) Ego functioning
SCID-I: Structured Clinical Interview for DSM-IV Axis I Disorders
SCID-II: Structured Clinical Interview for DSM-IV Axis II Disorders
SMART: (S) Specific, (M) measurable, (A) attainable, (R) relevant, (T) time-based
SUD: Substance use disorder
SPSS: Statistical Package for Social Science
QOL: Quality of life
